# Influence of Architected Core Topology on the Dynamic and Flexural Behaviour of Multi-Material Sandwich Structures

**DOI:** 10.3390/polym18060711

**Published:** 2026-03-14

**Authors:** Hilal Doğanay Katı, Muhammad Khan

**Affiliations:** 1 Faculty of Engineering and Natural Sciences, Department of Mechanical Engineering, Bursa Technical University, 16310 Bursa, Türkiye; 2 Centre for Life-Cycle Engineering and Management, Cranfield University, Bedford MK43 0AL, UK

**Keywords:** multi-material FDM, sandwich structures, experimental modal analysis, damping, natural frequencies, three-point bending

## Abstract

The integration of mechanics-based analysis and materials design procedures has become central to the development of multi-material structures with tailored mechanical and dynamic performance. In this study, the dynamic and flexural behaviour of multi-material FDM sandwich beams composed of PETG face sheets and an ABS core is experimentally investigated. Seven different infill patterns Grid, Line, Wavy, Honeycomb, Gyroid, Cubic, and Triangle were implemented in the core layer to assess their influence on damping and natural frequency behaviour. Experimental modal analysis was performed using impact testing to identify the first three vibration modes. Natural frequencies were extracted from Frequency Response Functions (FRFs), and modal damping ratios were determined using the half-power bandwidth method. The reliability of the damping results was evaluated through statistical analysis. Additionally, quasi-static three-point bending tests were conducted to assess flexural strength and load-carrying capacity. The results demonstrate that infill topology has a significant impact on both dynamic and mechanical responses. In particular, geometrically complex infill patterns exhibit enhanced stiffness, higher natural frequencies, and improved damping performance. Among the investigated designs, the Triangle infill exhibited the highest natural frequency values across the first three vibration modes (*f*_1_ ≈ 24.910 Hz, *f*_2_ ≈ 162.609 Hz, *f* ≈ 466.595 Hz), indicating its superior stiffness characteristics. In terms of damping behaviour, the Cubic infill showed the highest loss factor in the first vibration mode (0.0426), while the Line and Gyroid patterns exhibited the highest damping in the second (0.0439) and third modes (0.0354), respectively. Moreover, the force–displacement results revealed that the Triangle infill exhibited the highest load-bearing capacity, further confirming its superior structural stiffness among the investigated designs (SEA = 110.83 J/kg). These findings highlight the potential of multi-material FDM for designing polymer-based sandwich structures with tailored vibration and energy dissipation characteristics.

## 1. Introduction

Sandwich structures are widely preferred in many engineering applications including automotive, robotics, defence, aerospace, space and satellite structures, biomedical, and marine industries due to their ability to simultaneously provide high damping capacity, lightweight characteristics, and high flexural stiffness [1,2,3,4]. A detailed investigation of the vibration behaviour of such structures is of great importance, as an accurate understanding of their dynamic characteristics plays a critical role in ensuring the efficient, reliable, and long-lasting operation of mechanical systems. The mechanical and dynamic performance of sandwich structures strongly depends on the structural integrity formed by the interaction between the face sheets and the core. In particular, the core topology has a decisive influence on load transfer, local stability, energy absorption, and vibration behaviour [5,6,7]. By modifying the core geometry, the load-bearing behaviour, deformation characteristics, and vibration response of sandwich structures can be significantly altered. For this reason, architecturally controlled polymer cores have attracted increasing attention in recent years [8]. Advances in additive manufacturing technologies, especially the widespread adoption of the FDM method, have substantially enhanced the design flexibility of internal core architectures used in sandwich structures [9]. Through FDM, complex core geometries such as honeycomb, gyroid, lattice, triangular, auxetic, and biomimetic structures can be fabricated, enabling direct control over parameters including cell density, infill pattern, and material distribution [10,11,12,13,14,15]. Consequently, internal architectures that are difficult to achieve using conventional manufacturing methods can now be designed to meet specific mechanical and dynamic performance requirements.

Numerous studies in the literature have investigated the flexural, impact, and vibration behaviour of single-material 3D-printed beams [16,17,18,19,20,21,22]. The flexural and compressive strength as well as the energy absorption performance of honeycomb, gyroid, TPMS, triangular, auxetic, and lattice-based cores have been extensively examined [23,24,25,26,27,28]. For instance, Li and Wang [29] demonstrated that the flexural stiffness, load-carrying capacity, and energy absorption behaviour of 3D-printed sandwich composites are directly governed by core topology. Similarly, Sarvestani et al. [30] experimentally and numerically investigated damage mechanisms and energy dissipation capacity of metamaterial-core sandwich structures (cubic, octet, and Isomax) under flexural and impact loading, revealing that core topology plays a decisive role in stiffness, energy absorption, and multi-impact resistance. In another study, Liu et al. [31] examined the mechanical behaviour of lattice-core metal-polymer sandwich structures under bending loads and showed that BCC and FCC core topologies provide higher flexural stiffness and energy absorption due to more effective load transfer mechanisms.

With the development of multi-material FDM systems, sandwich structure performance can now be optimised not only through geometric design but also via material combinations [32,33,34]. In this context, a detailed investigation of their vibration behaviour is of great importance, as an accurate understanding of dynamic characteristics plays a critical role in ensuring the efficient, reliable, and long-term operation of mechanical systems [12,13,35,36]. Ahmed et al. [37] demonstrated that hybrid laminated composites composed of ABS and carbon fibre–reinforced PLA layers can achieve higher tensile strength and toughness compared to monolithic structures. Pinho and Piedade [38] investigated multi-material 3D-printed sandwich polymer structures with TPU cores and ABS, HIPS, or PMMA face sheets, showing that TPU cores enhance impact energy dissipation and stabilise mechanical behaviour even under ageing conditions. Edelen and Bruck [39] experimentally and analytically examined the influence of manufacturing parameters on the flexural behaviour and failure modes of multi-material polymer sandwich beams, indicating that damage mechanisms can be controlled through the selection of appropriate parameters. Similarly, Liu et al. [40] reported that printing parameters significantly affect interfacial bonding, flexural strength, and failure modes in soft–hard multi-material (PLA-TPU) sandwich beams. Waly et al. [41] further showed that compliant interlayers in PETG-TPC sandwich structures can deflect and arrest crack propagation, thereby improving fracture toughness and energy absorption. More recently, Darsin et al. [42] investigated multi-material PLA, PP, and ABS combinations and demonstrated that PLA-ABS layered structures provide the highest flexural strength without delamination.

Despite these advances, studies addressing the vibration behaviour of 3D-printed sandwich structures remain limited and are primarily focused on natural frequency variations [43]. Modal damping ratio, one of the most critical parameters for vibration control, has been considered in only a small number of studies [44,45]. In particular, research employing the half-power bandwidth method to evaluate modal damping is scarce, indicating that damping behaviour is still insufficiently represented in the literature [46,47,48]. Furthermore, most existing studies compare only a limited number of core geometries, and no systematic investigation has been reported that simultaneously examines a wide range of infill patterns, such as Grid, Line, Wavy Line, Honeycomb, Gyroid, Cubic, and Triangle, within a single framework. Recent investigations have highlighted the importance of architectural design in governing structural response. For example, Khan et al. [24] experimentally and numerically investigated the vibration behaviour of multi-material graded polymer plates, demonstrating that frequency-dependent viscoelastic material models play a critical role in accurately predicting natural frequencies and damping ratios. Eqbert et al. [49] compared the vibration characteristics of FDM-fabricated sandwich beams with BCC and TPMS (Gyroid, Diamond, and IWP) cores and showed that TPMS cores, particularly Gyroid and Diamond geometries, exhibit lower resonance amplitudes and more effective vibration damping performance. While these studies primarily focused on dynamic characteristics, Nasution et al. [50] evaluated the tensile performance of multi-material FDM sandwich structures made from PLA, ABS, and HIPS, revealing that the ABS-PLA-ABS configuration achieved the highest tensile strength and modulus due to improved interlayer bonding and material compatibility. These findings further align with recent work by Elhassan et al. [51], who demonstrated that slicer-level interfacial interlocking significantly improves flexural strength and energy absorption in multi-material FFF sandwich panels. This emphasizes that not only core topology but also interface architecture plays a decisive role in governing the mechanical response of additively manufactured sandwich structures.

Based on the current state of the literature, although significant progress has been made in understanding the mechanical and vibrational behaviour of additively manufactured sandwich structures, most existing studies either focus on single-material systems or investigate only a limited number of core geometries. Moreover, modal damping is rarely quantified using consistent frequency-domain approaches such as the half-power bandwidth method [52]. To date, no systematic investigation has simultaneously examined multiple infill topologies within a unified multi-material FDM framework while integrating experimental modal analysis, flexural stiffness extraction, and analytical validation. Therefore, the present study aims to experimentally evaluate the combined effects of infill topology and material combination on the dynamic and flexural response of PETG-ABS sandwich beams. Natural frequencies corresponding to the first three vibration modes were determined for seven different core infill patterns, and the modal loss factors of the first three modes were calculated using the half-power bandwidth method. The reliability of the obtained damping values was evaluated using statistical methods, including the coefficient of variation and correlation analysis. Additionally, three-point bending tests were conducted to assess the influence of core topology on flexural strength and load-carrying capacity. The results clearly demonstrate that infill topology is a critical design parameter in multi-material additively manufactured sandwich structures, governing not only stiffness and natural frequency but also energy dissipation behaviour.

## 2. Materials and Methods

In this study, the dynamic properties of PETG-ABS sandwich structures were investigated. This section outlines the manufacturing process and experimental procedures used to evaluate the vibration and bending responses of 3D-printed sandwich structures. The specimens were manufactured in the form of sandwich beams, and the work consisted of three main stages: (i) designing the sandwich configuration with PETG face sheets and an ABS core, (ii) fabricating the specimens using 3D printing, and (iii) conducting experimental modal analysis together with three-point bending tests to evaluate their mechanical and vibrational behaviour.

### 2.1. Manufacturing Process of the Sandwich Beams

**Figure 1:**
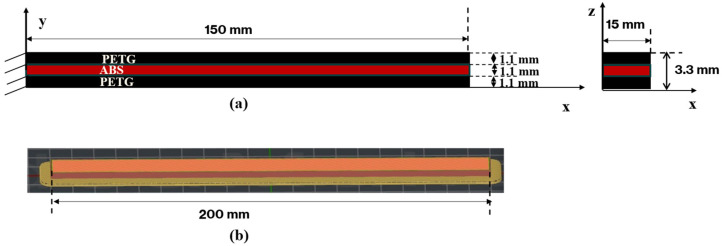
Schematic representation and cross-sectional view of the three-layer PETG-ABS-PETG sandwich beam, (**a**) illustrating the layer sequence, material distribution, and (**b**) overall beam geometry.

Composite beams with a sandwich structure were produced using a combination of polyethene terephthalate glycol (PETG) and Acrylonitrile-butadiene-styrene (ABS) materials. As shown in Figure 1, the beam has a total length of 200 mm, a width of 15 mm, and a total thickness of 3.3 mm. The structure consists of three layers: 1.1 mm of PETG on top, 1.1 mm of ABS in the middle, and 1.1 mm of PETG on the bottom, each layer being of equal thickness. The material forming the middle layer (core) of the produced beams is ABS, and this region was manufactured with seven different infill patterns. The infill patterns used in the middle layer were determined as Grid, Line, Wavy, Honeycomb, Gyroid, Cubic, and Triangle. The outer layers on the upper and lower surfaces of the beam were produced entirely from PETG material. This structure is illustrated in Figure 1, where the line infill structure of PETG is employed in the upper and lower layers, while the infill patterns in the ABS part of the middle layer are modified to achieve different structural behaviours.

**Figure 2:**
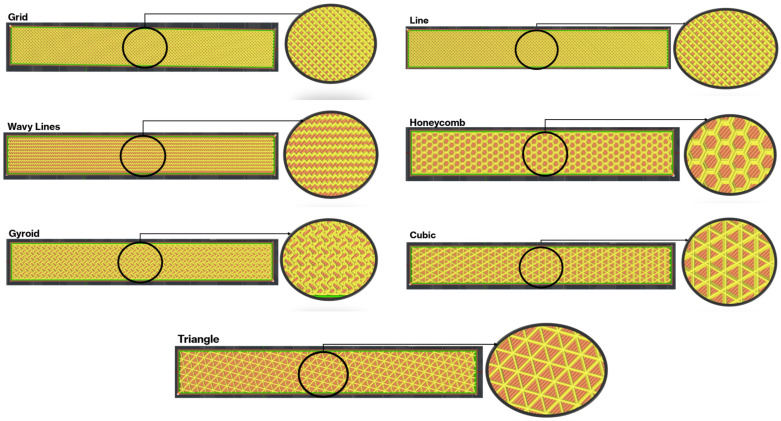
Slicer-based view and close-up internal structure details of different infill patterns used in the middle layer of the sandwich composite beam.

During the production phase, a 3D CAD model of the beam with a thickness of 1.1 mm was first created using SolidWorks© (2024) and saved in STL format. This model was then transferred to the Ideamaker slicing software (version of 5.1.4), where the printing parameters were adjusted. The G-codes were generated by the slicer and used directly as input for the Raise3D Pro printer. The hardware produced the test specimen in the required dimensions. All samples were manufactured using a 3D printer, with the basic parameters maintained constant throughout the printing process. The infill density was set to the maximum value permitted by the slicer for each infill topology. Although this setting is referred to as 100% infill, the internal architecture still differs significantly depending on the selected infill pattern. All additional printing parameters were specified following the settings provided in Table 1. All specimens were printed with the beam longitudinal axis aligned with the printing direction to minimise anisotropic effects. The PETG face sheets and ABS core were fabricated through a single continuous printing process, ensuring direct interfacial bonding without the need for additional adhesives. The layer height was set to 0.2 mm for both PETG and ABS throughout the printing process. The sandwich structure was manufactured sequentially within a single uninterrupted build: the bottom PETG face sheet (1.1 mm) was first deposited layer-by-layer, followed immediately by the ABS core region (1.1 mm) with the selected infill topology, and finally the top PETG face sheet (1.1 mm). Material switching was performed automatically by the dual-extrusion system without cooling interruption, ensuring that the previously deposited layer remained within a thermally active state during subsequent deposition. No adhesive or post-processing treatment was applied at the interface. This continuous deposition strategy was adopted to promote interfacial diffusion and mechanical interlocking between PETG and ABS. Additionally, internal structure images for each infill pattern are presented in Figure 2, clearly illustrating the geometric differences between the samples.

The selection of the seven infill patterns was not arbitrary but was designed to represent a broad spectrum of internal architectures commonly employed in material extrusion-based additive manufacturing. The chosen patterns include stretch-dominated structures (e.g., Grid, Cubic), bending-dominated cellular configurations (e.g., Honeycomb, Triangle), and triply periodic minimal surface (TPMS) architectures (e.g., Gyroid), which are known for their quasi-isotropic mechanical response and enhanced energy dissipation characteristics. Additionally, Line and Wavy patterns were included to represent simpler directional infill strategies frequently used in practical FDM applications. This diversity allows systematic comparison of architected core topologies with fundamentally different load transfer mechanisms, stiffness characteristics, and deformation modes within a unified multi-material sandwich framework.

### 2.2. Test Method for Vibration Behaviour

**Figure 3:**
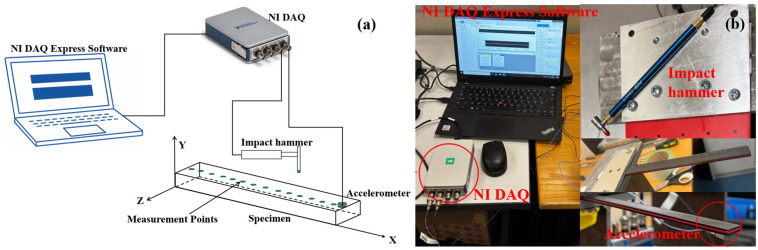
(**a**) The schematic diagram of the experimental setup; (**b**) Test equipment of EMA.

After the specimens were produced using a 3D printer, Experimental Modal Analysis (EMA) was conducted to evaluate the vibration performance of the sandwich composite beam. The specimen dimensions were selected to satisfy both flexural testing and modal identification requirements. Specifically, the geometry ensured bending-dominated behaviour under three-point loading and clear separation of the first three vibration modes within the measurable frequency range, while remaining compatible with the printer build volume and experimental fixtures. The produced beams had a total length of 200 mm, with a 50 mm end section fixed as a cantilever. Thus, the free length subjected to the vibration test was 150 mm. Although a fixed–free boundary condition was assumed in the experimental setup, perfect clamping is difficult to achieve in practice. A small degree of boundary compliance is therefore unavoidable and is expected to mainly influence the first vibration mode. The experimental setup (Figure 3b) consisted of data acquisition equipment, including an NI 9234 module and an NI 9174 chassis (National Instruments, London, UK), a modal impact hammer (PCB 086E80) with a sensitivity of 22.5 mV/N, a miniature piezoelectric accelerometer (PCB 352A21, with a sensitivity of 10 mV/g), and the sandwich composite beam specimen [52]. The accelerometer was placed on the bottom layer of the beam near the free end. Both the accelerometer and the impact hammer were connected to the vibration acquisition device, and the acceleration response data generated by the vibration were transferred to the computer through the data acquisition software (and NI DAQ Express v5.1). The mass of the accelerometer is small compared to the total mass of the beam and was kept constant for all tests; therefore, its influence on the comparative modal analysis is considered negligible. Subsequently, the beam was impacted individually at 15 measurement points, spaced approximately 10 mm apart. along the beam, and the accelerometer responses were recorded in the time domain. Data were sampled at 25.6 kHz and processed using the Welch method with a 50% overlap, yielding a frequency resolution of approximately 0.78 Hz. The schematic diagram of the experimental setup is presented in Figure 3a. The collected time-domain data were transformed into the frequency domain by using MATLAB R2024a, and the 15 FRFs were plotted together. To evaluate the frequency and damping characteristics in the thickness direction, the average of these 15 FRFs was calculated to reduce random noise, inconsistent hammer strikes, and local structural variations.

The loss factor (*η*) of each mode was determined using the half-power bandwidth method, which is a standard frequency-domain approach in modal analysis. For a single-degree-of-freedom resonance peak, the loss factor can be expressed as:

(1) $$\eta =\frac{f_{b}-f_{a}}{f_{r}}$$

where $f_{r}$, $f_{a}$ and $f_{b}$ is resonance (peak) frequency, lower and upper half-power frequencies, respectively, defined as the frequencies at which the amplitude drops by 3 dB relative to the peak amplitude.

**Figure 4:**
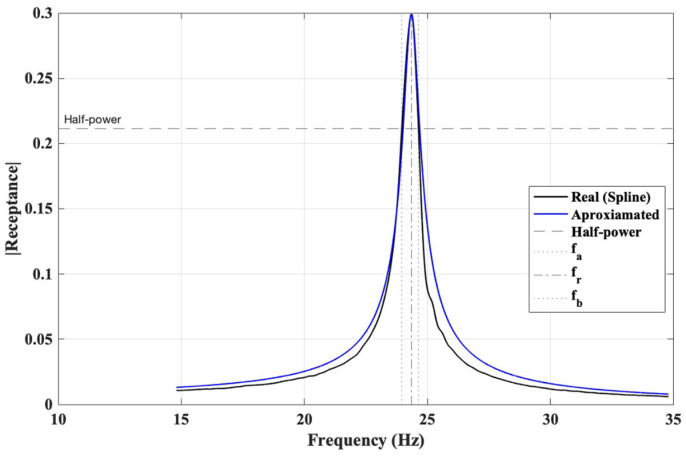
Half-Power based damping identification from experimental FRF data.

The half-power points were obtained from the magnitude of the FRF. To ensure the precise extraction of the bandwidth, the FRF curves were interpolated using spline smoothing, and the 3 dB drop was computed numerically rather than relying on discrete measurement points (Figure 4). This approach reduces discretisation error, particularly near sharp resonance peaks. It should be noted that the half-power bandwidth method assumes lightly damped single-degree-of-freedom behaviour around each resonance peak. The accuracy of the method may decrease in cases of very low damping ratios or closely spaced modes, where modal overlap can lead to ambiguity in identifying the −3 dB bandwidth limits. In addition, measurement resolution and noise sensitivity may influence the precision of damping estimation. However, in the present study, the first three vibration modes were sufficiently separated in frequency, and the resonance peaks were clearly identifiable, allowing reliable application of the half-power bandwidth approach.

### 2.3. Three-Point Bending Test

**Figure 5:**
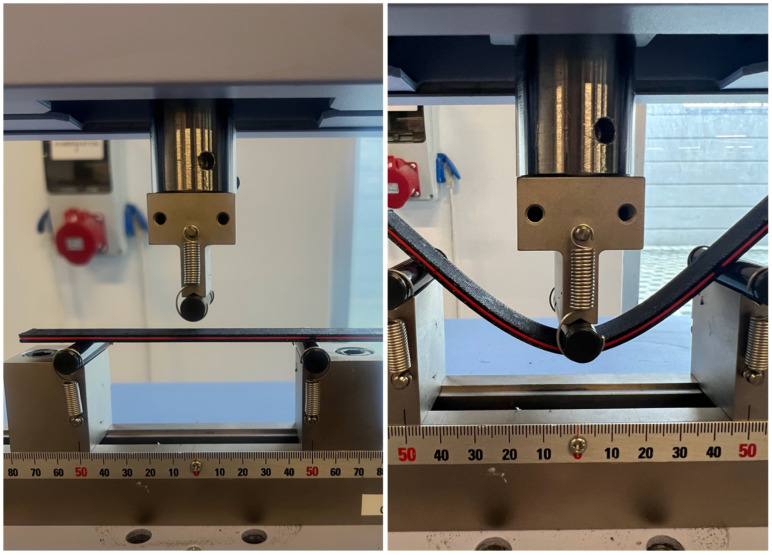
Three-point bending test setup and deformation of the sandwich beam under loading.

Three-point bending tests were performed to investigate the flexural response of sandwich composite beam specimens. For this reason, the tests were performed at room temperature using a Shimadzu AGS-J model universal testing machine. The loading rate was selected as 2 mm/min to ensure quasi-static loading conditions. In the test setup, the specimen was placed on the lower supports with a clearance of 100 mm (Figure 5) and the load was applied from the central point with the upper compression head. During the test, the force and displacement data were recorded by the internal measurement system (TRAPEZİUM X software version 14.0) of the device and used to generate force-displacement curves. Each specimen was tested only once, due to the high repeatability of the printing process; the results are therefore interpreted in a comparative rather than absolute sense.

In addition to the force-displacement response, the apparent flexural strength ($\sigma_{f}$) of the sandwich beams was calculated from the maximum load obtained during the three-point bending tests using the classical beam theory expression:

(2) $$\sigma_{f}=\frac{3FL}{2bh^{2}}$$

where *F* is the maximum load, *L* is the support span, *b* is the specimen width, and *h* is the specimen thickness.

#### 2.3.1. Specific Energy Absorption (SEA) Calculation

The absorbed energy (EA) was calculated as the area under the force-displacement curve obtained from the three-point bending tests, up to the maximum recorded displacement. The absorbed energy was computed as:

(3) $$EA=\int_{0}^{S_{max}}F(S)\;dS$$

where *F* is the applied force and *S* is the corresponding displacement. The specific energy absorption (SEA) was then determined by normalising the absorbed energy with respect to the specimen mass:

(4) $$SEA=\frac{EA}{m}$$

where *m* is the measured mass of the sandwich beam specimen.

The mass of each sandwich beam specimen was measured prior to mechanical and modal testing using a precision digital balance with a resolution of 0.001 g. The measured masses (Table 2) were used to calculate the mass per unit length of the cantilever beams, which is required for the analytical estimation of natural frequencies based on Euler-Bernoulli beam theory. SEA values were determined using the measured specimen masses corresponding to a beam length of 200 mm. Since the specimen mass constitutes an input parameter for the analytical model rather than an experimental outcome, the measured values are reported here for completeness and reproducibility.

#### 2.3.2. Analytical Frequency Estimation Based on the Three-Point Bending Test

The flexural modulus *E* (MPa) is determined from the slope of the load–deflection curve (Δ*F*/Δ*S*) within the linear elastic region:

(5) $$E=\frac{L^{3}}{4bh^{3}}\left(\frac{\Delta F}{\Delta S}\right)$$

where *b* is the specimen width, *h* is the specimen thickness, *S* is the mid-span deflection, and *L* is the support span in the three-point bending test.

The mass per unit length required in Equation (6) was calculated using the experimentally measured specimen masses listed in Table 2.

(6) $$\mu =\frac{m}{L_{b}}$$

where *μ* is the mass per unit length (kg/m), *m* is the measured mass of the specimen (kg), and $L_{b}$ is the free length of the cantilever beam (150 mm).

Using the flexural modulus obtained from the three-point bending tests and the measured mass distribution, the natural frequencies of the cantilever sandwich beams were analytically estimated based on Euler–Bernoulli beam theory:

(7) $$f_{n}=\frac{\beta_{n}^{2}}{2\pi L_{b}^{2}}\sqrt{\frac{EI}{\mu }}\;\;\;\;\;\;\;\;\;\;\;\;\;\;\;\;\;(n=1,2,3)$$

where $f_{n}$ is the nth natural frequency (Hz), *E* is the flexural modulus obtained from three-point bending tests. For a cantilever beam, the modal constants are $\beta_{1}=1.875,{\;\beta }_{2}=4.694,{\;\beta }_{3}=7.855$.

Although sandwich beams may exhibit shear deformation due to the presence of a relatively compliant core, the present specimens possess a high slenderness ratio (L/h ≈ 60), indicating bending-dominated behaviour. For such geometries, shear deformation effects are expected to be relatively small in the lower vibration modes considered. The analytical model based on Euler–Bernoulli beam theory was therefore adopted for simplicity and for comparative validation of experimental trends rather than for high-fidelity prediction of absolute natural frequency values. Consequently, the neglect of Timoshenko shear deformation effects is considered justified within the scope of the present study.

Theoretical natural frequencies were calculated using the Euler-Bernoulli cantilever beam model based on flexural modulus values obtained from three-point bending tests. Percentage error is defined as;

(8) $$\%Error=\frac{\left|f_{theo}-f_{EMA}\right|}{f_{EMA}}\times 100$$

### 2.4. Statistical Analysis

To quantitatively evaluate the influence of infill geometry on the modal behaviour of the sandwich composite beams, a statistical analysis was conducted using the coefficient of variation (*CV*) and Pearson correlation. The *CV*, defined as the ratio of the standard deviation (*σ*) to the mean (*μ*) (Equation (9)), was used to assess the relative dispersion of the natural frequency and damping values across the seven infill patterns:

(9) $$CV=\frac{\sigma }{\mu }$$

## 3. Results and Discussion

**Figure 6:**
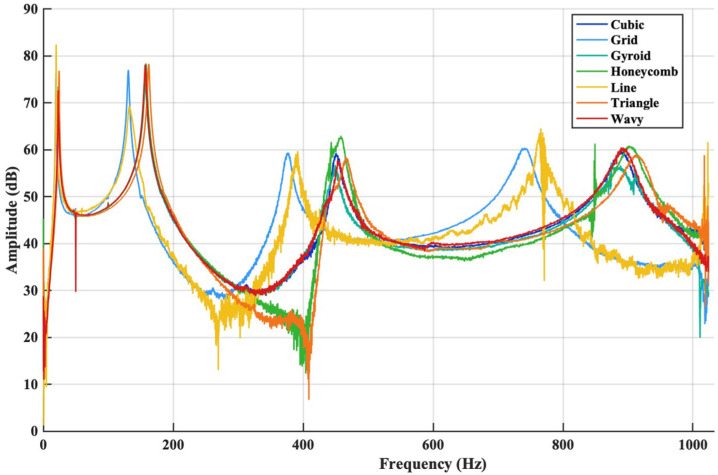
FRF behaviours of sandwich beams according to different infill patterns.

**Figure 7:**
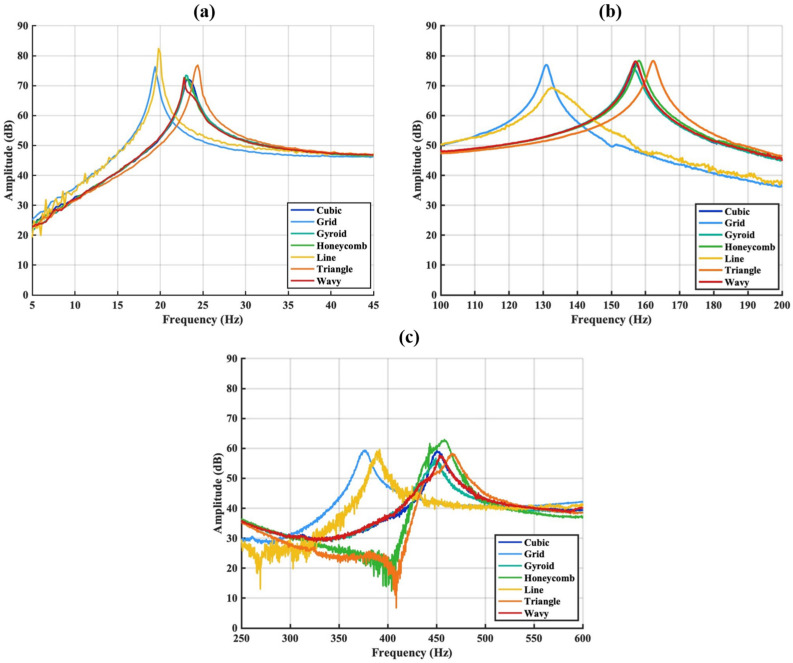
Comparison of the FRF magnitudes of sandwich composite beams with different infill patterns in the first (**a**), second (**b**) and third (**c**) mode frequency bands.

The FRFs of the sandwich composite structure were examined by keeping the accelerometer fixed at the free end of the beam while varying the excitation position of the impact force. By analysing the first three bending modes of the sandwich composite structure, the frequency and damping characteristics in the thickness direction were evaluated for different infill patterns used in the middle layer. The modal behaviour of sandwich structures with different infill patterns has been comprehensively evaluated by examining the natural frequency values presented in Table 3 and the FRF results given in Figure 6 and Figure 7. According to Table 3 the Grid and Line patterns exhibit the lowest natural frequencies across all modes, indicating that these patterns result in lower structural rigidity in the sandwich structure. This is clearly confirmed by the low-frequency behaviour, peaking at approximately 19–20 Hz in the first mode (Figure 7a). In contrast, more complex cellular geometries such as Honeycomb, Gyroid, Cubic, and Wavy, as well as patterns with sharp and closed surfaces such as Triangle, were observed to increase the first mode frequencies to the 22–25 Hz range. These results indicate that these patterns contribute to the increase in the first mode natural frequency by enhancing the bending rigidity of the sandwich structure.

In the broadband FRF graph (Figure 6), when the collective behaviour of all modes is evaluated together, it is clear that the infill pattern has a strong dynamic effect on the sandwich structure. Line and Grid infill structures, which have simpler geometric patterns, exhibit higher amplitude variations over a wide frequency range, indicating a more compliant internal structure with reduced effective stiffness. In contrast, infill patterns such as Triangle, Honeycomb, Gyroid, and Cubic show a more stable amplitude distribution and resonance responses shifted toward higher frequencies in the higher modes, reflecting an overall increase in sandwich structure rigidity. The Wavy pattern exhibits pronounced amplitude fluctuations within specific frequency bands, suggesting a different vibration energy distribution mechanism compared to the other infill geometries, which distinguishes its dynamic response characteristics.

Upon detailed examination of the second mode region (Figure 7b), it is observed that the Grid and Line patterns are again located at the lowest frequency values (≈131–132 Hz). In contrast, the Honeycomb, Gyroid, Cubic, Triangle, and Wavy patterns cluster in the 156–162 Hz range, indicating that these patterns also form structures with higher rigidity in their second vibration modes. In particular, the fact that the Triangle pattern has the highest natural frequency, approximately 162 Hz, in the second mode makes the rigidity-enhancing effect of this geometry within the sandwich structure more pronounced. This trend becomes even more pronounced in the third mode region. As shown in Figure 7c, the Grid and Line patterns exhibit the lowest natural frequencies (≈375–390 Hz) in the third mode, whereas the Honeycomb, Gyroid, Cubic, and Wavy patterns are concentrated in the 448–458 Hz range. The Triangle pattern again provides the highest value in the third mode at ≈466 Hz, indicating that this pattern offers the highest structural rigidity throughout all modes within the sandwich structure.

Both the natural frequency values and the amplitude-frequency relationship obtained from the FRF curves (Figure 6 and Figure 7) demonstrate that infill geometry is a decisive parameter in modal characteristics. Patterns with higher geometric complexity and cellular density (particularly Triangle, Honeycomb and Cubic) exhibit higher mode frequencies and more stable vibration behaviour, while patterns with more open cells and linear designs (Grid and Line) produce lower stiffness and lower natural frequencies. These findings confirm that infill geometry is a critical design variable in optimising vibration performance.

**Figure 8:**
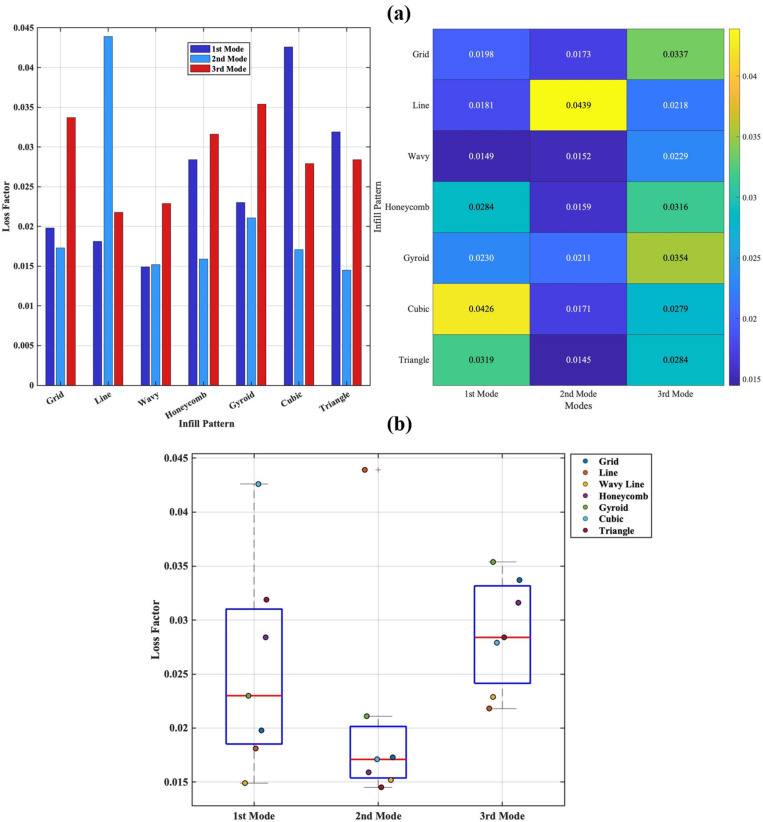
Comparison of modal loss factor for different infill patterns: (**a**) Grouped bar chart combined with a heatmap illustrating mode-dependent variation; (**b**) Boxplot with scatter overlay showing statistical distribution and outliers.

The two graphs presented in Figure 8 comprehensively demonstrate both comparatively and statistically the effect of producing the middle layer with different infill patterns on the modal damping behaviour of a three-layer sandwich composite beam consisting of an ABS core and PETG surface layers. The grouped bar graph and heat map (Figure 8a) clearly show the quantitative effect of infill patterns on the modes. In the first mode, the Cubic, Honeycomb, and Triangle patterns produce higher loss factors, while the Grid, Line, and Wavy patterns remain at low levels. In the second mode, the Line pattern clearly stands out from the other patterns by reaching a distinct maximum. In the third mode, the Gyroid, Honeycomb, and Grid patterns exhibit higher damping performance. On the other hand, Figure 8b reveals the statistical properties of the same data and confirms the reliability of the trends observed in Figure 8a. The wide distribution of loss factor values in the first mode suggests that the infill geometry has a significant impact on low-frequency vibrations. High values for patterns such as Cubic and Triangle are evident at outlier or upper quartile levels, while Grid and Wavy patterns are seen to be close to the lower quartile values. The narrower range of values in the second mode indicates that the infill pattern has a limited effect on this mode; however, the single high value of the Line pattern indicates that local deformation effects associated with the mode shape can significantly influence the damping behaviour. The wider distribution in the third mode confirms that both the viscoelastic characteristics of the material and the infill topology play a more prominent role at high frequencies.

The observed differences in modal loss factor among the infill topologies can be physically attributed to variations in deformation mechanisms and internal strain distribution within the architected core. In open-cell, line-dominated structures such as Line and Grid, load transfer occurs primarily through axial stretching of relatively straight filaments, leading to more uniform strain distribution and limited internal friction. Consequently, energy dissipation remains comparatively low in these designs [8]. In contrast, closed or highly interconnected geometries such as Triangle, Cubic, and Gyroid promote localized bending, torsion, and stress concentration at junction regions. These complex deformation modes increase micro-scale friction between filament interfaces and enhance viscoelastic energy dissipation within the ABS core material. TPMS-based structures such as Gyroid, in particular, exhibit continuous curved surfaces that induce distributed shear deformation, which can further amplify internal damping mechanisms. Therefore, the variation in loss factor is not solely a geometric effect but results from the interaction between core topology, deformation mode, and the intrinsic viscoelastic behaviour of the polymer material [49].

**Figure 9:**
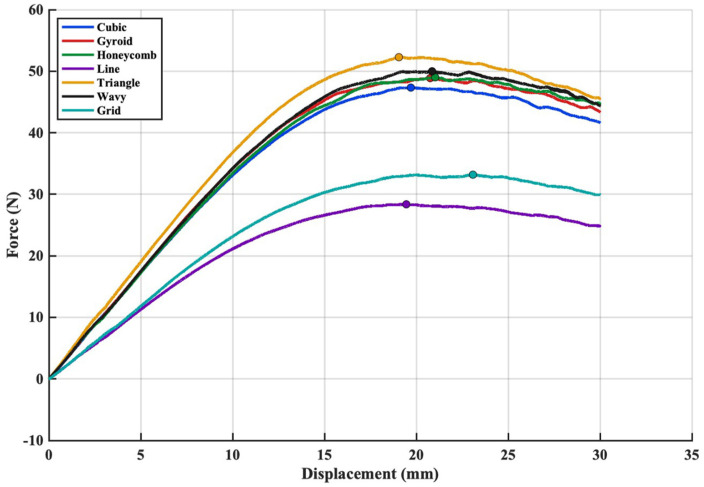
Force-displacement curves of the sandwich beams with different core configurations under bending tests. The circle markers indicate the maximum force points.

The flexural responses of the specimens were compared by presenting all force–displacement curves on the same graph (Figure 9). The curves were restricted to the first 30 mm of displacement because deformation at higher displacement levels caused material accumulation beneath the loading nose, leading to an artificial increase in force and not reflecting the actual flexural behaviour of the infill structures. From the analysis of the load-deflection curve, it is shown that the Triangle core reaches the highest load among all infill patterns. This indicates that its internal geometry distributes the bending forces more effectively than the other configurations. The quicker rise in force and the later reduction in stiffness suggest that the triangular cell layout provides more stable load-carrying paths, allowing the specimen to endure higher stress levels before local buckling begins. Similar behaviour was also noted by Chahardoli and Attar [23], who reported that triangular core designs offered better resistance to deformation compared with other core shapes. During the three-point bending tests, no interlayer separation or debonding was observed between the PETG face sheets and the ABS core, indicating that the layers adhered effectively to one another during the direct multi-material printing process despite the absence of any additional adhesive. The absence of interlayer separation between PETG and ABS observed in the present study is consistent with previous multi-material FDM investigations reporting that certain thermoplastic combinations can achieve sufficient interfacial bonding under compatible processing conditions. For instance, Darsin et al. [42] reported that PLA-ABS specimens did not exhibit delamination during three-point bending, whereas other material combinations such as PLA-PP and PP-ABS showed significant interfacial separation due to inadequate bonding and thermal incompatibility. These findings suggest that interfacial integrity in multi-material FDM systems strongly depends on material compatibility and printing temperature control.

In addition to the force-displacement response, the apparent flexural strength values calculated from the maximum loads further support the observed bending behaviour. The calculated apparent flexural strength values are consistent with the trends observed in the force–displacement curves. The Triangle infill exhibited the highest flexural strength among all configurations, indicating a more efficient load transfer under bending. In contrast, patterns such as Grid and Line showed comparatively lower strength due to their less interconnected internal architecture. These results confirm that the bending performance of multi-material sandwich beams is strongly influenced by the selected core topology.

The bending modulus E values obtained from three-point bending tests were used to theoretically calculate the first three natural frequencies for the fixed-free beam using the Euler-Bernoulli approach and compared with the EMA results. The comparison results show that the theoretical model generally captures the frequency trends correctly: in all infill patterns, the theoretical frequencies were close to the values measured by EMA; in particular, the error levels were low in the 2nd and 3rd modes. The average absolute error was found to be approximately 2.34% for the 2nd mode and 1.50% for the 3rd mode, while the error was higher in the 1st mode, averaging 10.62%. The systematic overestimation of theoretical predictions in the 1st mode can be attributed to several reasons, including shear deformation in sandwich beams (Timoshenko effect), boundary compliance under fixed conditions, interaction between multi-material layers, and the E value derived from static bending not fully representing the effective stiffness under dynamic conditions. In contrast, the significant increase in compliance in the 2nd and 3rd modes indicates that the model based on the measured mass distribution (μ) and bending stiffness (EI) better represents the dominant behaviour in high modes. In conclusion, this validation study demonstrates that the stiffness parameters derived from three-point bending mechanically support the natural frequency changes observed using EMA, and that the infill topology guides the dynamic response through its stiffness. The deformation and load transfer characteristics of the different core topologies can be interpreted based on established cellular solids theory, which distinguishes between stretch-dominated and bending-dominated architectures [53]. Stretch-dominated configurations such as Triangle and Cubic promote axial load transfer along interconnected struts, leading to enhanced stiffness and delayed buckling, whereas bending-dominated structures deform primarily through flexural mechanisms of cell walls. Similarly, TPMS-based architectures such as Gyroid exhibit distributed deformation due to their continuous curvature-driven geometry [54,55] which influences both stiffness and damping behaviour. These complex deformation modes—including localized bending and torsion at junction regions—increase micro-scale internal friction and enhance viscoelastic energy dissipation within the ABS core material [24].

The CV values were calculated as 0.078, 0.081, and 0.077 for the 1st, 2nd and 3rd natural frequencies, respectively. Although the 2nd mode exhibits the highest CV, this variation is primarily attributed to the broader frequency scale of that mode rather than a stronger geometric sensitivity. When interpreted in conjunction with the absolute modal frequencies, the results confirm that the 1st and 3rd modes are more responsive to changes in infill geometry, consistent with their higher dependence on structural stiffness. Pearson correlation analysis further revealed very strong positive correlations between all mode pairs (r > 0.97). This demonstrates that stiffness variations caused by infill geometry follow a coherent trend across all vibration modes. In other words, infill designs that increase stiffness in the 1st mode tend to sustain this behaviour in the higher modes as well.

For the damping characteristics, the CV values were determined as 0.355, 0.486, and 0.175 for the 1st, 2nd and 3rd modes, respectively. The highest variability observed in the second mode indicates that intermediate-frequency energy dissipation is more sensitive to differences in infill topology. Conversely, the third mode exhibited the lowest dispersion, suggesting that damping mechanisms at higher frequencies are more uniform among the different infill designs. Pearson correlation analysis further showed that damping in the first and third modes is moderately correlated (r = 0.695), while the 2nd mode demonstrated negligible or negative correlations with the others (r = −0.014 with Mode 1 and r = −0.173 with Mode 3). This finding suggests that the second mode is governed by a distinct deformation and strain distribution, resulting in different damping behaviour compared to the low- and high-frequency modes.

The combined statistical findings confirm that infill geometry is a dominant design parameter influencing both the natural frequencies and damping characteristics of sandwich composite beams. While modal frequencies exhibit a highly consistent geometric sensitivity across all modes, the damping behaviour shows mode-dependent variability, particularly pronounced in the intermediate-frequency range. These results reinforce the conclusion that infill topology plays a critical role in tailoring the dynamic performance of additively manufactured sandwich structures.

## 4. Conclusions

This study experimentally investigated the dynamic and flexural behaviour of multi-material FDM sandwich beams composed of PETG face sheets and an ABS core incorporating seven different infill topologies. By combining EMA with three-point bending tests, the influence of core architecture on natural frequency, damping behaviour, and structural stiffness was systematically evaluated. The modal analysis results showed that infill topology has a pronounced effect on the vibration characteristics of the sandwich beams. Infill patterns with higher geometric complexity, particularly the Triangle and Cubic configurations, consistently exhibited higher natural frequencies across the first three modes, indicating enhanced structural rigidity. Modal damping values (Loss factor) obtained using the half-power bandwidth method further revealed that damping behaviour is strongly dependent on infill architecture, with statistically distinguishable trends confirmed through coefficient of variation and correlation analyses.

The flexural test results supported the modal findings, demonstrating that core topology has a significant influence on load-carrying capacity and bending resistance. In particular, Triangular infill structures provided the highest flexural strength, highlighting the efficiency of closed and well-connected load paths within the core. Importantly, no interlayer separation was observed between the PETG face sheets and the ABS core during bending, confirming the effectiveness of direct multi-material FDM deposition without the use of additional adhesives.

The natural frequencies estimated from flexural stiffness show the same ranking trend as the experimental modal analysis, confirming that the increase in bending stiffness due to infill topology directly contributes to the observed frequency enhancement. The higher prediction error observed in the first mode is partly attributed to shear deformation and boundary compliance, while the improved agreement in higher modes confirms the model’s suitability for trend analysis. From a design perspective, Triangular infills are preferable when maximum stiffness and load-carrying capacity are required, whereas Triangle, cubic and gyroid infills offer superior damping characteristics. These insights provide practical guidance for vibration-oriented design of multi-material FDM sandwich structures.

Taken together, the results clearly demonstrate that infill geometry governs not only the static stiffness but also the positioning of natural frequencies, resonance amplitudes, and the spectral distribution of vibration energy. Accordingly, infill topology must be regarded as a primary and decisive design parameter for optimising the vibration performance of multi-material additively manufactured sandwich structures. These findings provide practical guidance for designing vibration-optimised lightweight components and highlight the significant potential of multi-material FDM in engineering sandwich structures with tailored dynamic performance. Future work may extend the present approach to higher vibration modes, fatigue behaviour, and alternative material combinations to further enhance the applicability of the proposed design strategy.

## Figures and Tables

**Table 1:** Printing parameters of ABS and PETG materials.

Parameter	ABS	PETG
Nozzle temperature (°C)	240	240
Nozzle diameter (mm)	0.4	0.4
Filament diameter (mm)	1.75	1.75
Build plate temperature (°C)	100	80
Printing speed (mm/s)	60.0	45.0
Melting cooling fan	Turned off	Turned on

**Table 2:** Energy absorption (EA), Specific Energy Absorption (SEA) and Flexural Strength (FS) of sandwich beam specimens.

Infill Pattern	EA (J)	Mass (kg)	SEA (J/kg)	FS (MPA)
Grid	0.735	0.0103005	71.32	30.47
Line	0.640	0.0098915	64.75	26.056
Wavy	1.100	0.0103854	105.90	45.911
Honeycomb	1.078	0.0103494	104.18	44.996
Gyroid	1.082	0.0104381	103.66	44.881
Cubic	1.047	0.0103473	101.22	43.466
Triangle	1.157	0.0104393	110.83	48.007

**Table 3:** Comparison of experimentally measured (EMA) and theoretically predicted natural frequencies (Hz) and percentage errors (%) for cantilever sandwich beams. f_1_, f_2_, and f_3_ represent the first, second, and third natural frequencies, respectively.

Infill Pattern	f_1_,_EMA_	f_1_,_Theo_	Error_1_	f_2_,_EMA_	f_2_,_Theo_	Error_2_	f_3_,_EMA_	f_3_,_Theo_	Error_3_
Grid	19.398	21.06	8.57	131.008	132.01	0.76	375.637	369.67	1.59
Line	19.852	21.23	6.94	132.277	133.03	0.57	389.734	372.52	4.42
Wavy	22.810	25.62	12.32	157.022	160.59	2.27	452.290	449.70	0.57
Honeycomb	22.849	26.09	14.18	158.089	163.54	3.45	457.803	457.96	0.03
Gyroid	23.065	25.72	11.51	156.503	161.22	3.01	448.157	451.47	0.74
Cubic	23.145	25.45	9.96	157.197	159.50	1.47	450.513	446.64	0.86
Triangle	24.496	27.16	10.88	162.338	170.22	4.86	465.894	476.68	2.32

## Data Availability

The data presented in this study is available on request from the corresponding author.
